# Physical rehabilitation versus no physical rehabilitation after total hip and knee arthroplasties: Protocol for a pragmatic, randomized, controlled, superiority trial (The DRAW1 trial)

**DOI:** 10.12688/f1000research.50814.1

**Published:** 2021-02-25

**Authors:** Troels Mark-Christensen, Kristian Thorborg, Thomas Kallemose, Thomas Bandholm

**Affiliations:** 1 Department of Rehabilitation, Centre of Health, Regional Municipality of Bornholm, Rønne, Bornholm, Denmark; 2 Physical Medicine and Rehabilitation Research - Copenhagen (PMR-C), Copenhagen University Hospital - Amager and Hvidovre, Copenhagen, Denmark; 3 Department of Physical Therapy and Occupational, Copenhagen University Hospital - Amager and Hvidovre, Copenhagen, Denmark; 4 Department of Orthopedic Surgery, Copenhagen University Hospital - Amager and Hvidovre, Copenhagen, Denmark; 5 Department of Clinical Medicine, University of Copenhagen, Copenhagen, Denmark; 6 Department of Clinical Research, Copenhagen University Hospital – Amager and Hvidovre, Copenhagen, Denmark

**Keywords:** Total Joint Replacement, Total Hip Arthroplasty, Total Knee Arthroplasty, Rehabilitation, Telerehabilitation.

## Abstract

**Background: **Following total hip- and knee arthroplasty (THA and TKA), post-discharge physical rehabilitation is common practice, but varies significantly regarding content, duration, intensity and mode of delivery. Recent systematic reviews have found home-based rehabilitation to be as good as outpatient rehabilitation in terms of pain and physical function. We therefore wonder if physical rehabilitation “works” at all when compared to no physical rehabilitation after THA and TKA – “no rehabilitation” defined as no prescribed therapeutic rehabilitation exercises. The purpose of this trial is to compare the effectiveness of home-based telerehabilitation, home-based rehabilitation and no physical rehabilitation following THA and TKA.

**Methods:** This pragmatic, randomized controlled trial will include 168 patients following discharge after THA or TKA, in Bornholm Denmark. Patients will be randomized into one of the three 6-week rehabilitation strategies: home-based telerehabilitation, home-based rehabilitation or no physical rehabilitation. The trial is designed as a superiority trial to test the hypothesis that rehabilitation (home-based telerehabilitation and home-based rehabilitation) is superior to no physical rehabilitation. The primary outcome will be the hip disability and osteoarthritis outcome score (HOOS)/ the knee injury and osteoarthritis outcome score (KOOS)-subscale: function of daily living at first follow-up (end of the 6-weeks' intervention). Additional follow-ups are scheduled at 3 and 12 months. Outcome assessors and data analysts are blinded to group allocation.

**Conclusions:** Knowledge about the effectiveness of the three investigated rehabilitation strategies will help guide the future organization of post-discharge rehabilitation after THA and TKA.

**Trial registration:** Clinicaltrials.gov NCT03750448 (23/11/2018)

## Background

Osteoarthritis is the leading cause of disability and pain among adults ^1^, and when non-surgical treatment does not adequately relieve symptoms, joint arthroplasty is considered. Total hip and knee arthroplasty (THA and TKA, respectively) are considered effective for end-stage osteoarthritis in restoring function of the affected joint, relieve symptoms and improving quality of life ^2– 4^. Post-discharge physical rehabilitation following THA and TKA is widely promoted to restore function and mobility of the affected joint ^5, 6^. However, the practices of post-discharge physical rehabilitation following THA and TKA vary significantly in content, duration and intensity, where the current practices are ranging from a single session of professional advice while still in hospital to several weeks of intensive in- or outpatient physical rehabilitation or home-based interventions ^5– 11^.

### Context placement

Several systematic reviews and meta-analyses of randomized controlled trials (RCTs) have examined the effectiveness of different types of post-discharge rehabilitation delivery strategies following THA and TKA ^5, 12– 14^. Generally, home-based rehabilitation after initial instruction is as effective as more closely supervised outpatient rehabilitation in the long term ^5, 7, 13– 16^, even in patients at risk of a poor outcome after TKA ^17^. When compared to no or minimal physical rehabilitation, physiotherapy exercises seem superior for improvements in physical function, pain and range of motion up to 3–6 months following TKA ^7^. We recently undertook a systematic review ^18^ of RCTs that compared physical rehabilitation strictly to a “no physical rehabilitation” comparator on patient-reported outcomes for function and pain and identified only two trials ^19, 20^. The review was inconclusive, and we – along with others ^7^ – call for sufficiently powered trials that investigate physical rehabilitation after THA and TKA against no physical rehabilitation comparators.

### Information gain

Traditionally, the type of physical rehabilitation exercise and degree of supervision are regarded as important factors for postoperative recovery of functional performance; however, this does not seem to be the case. Rehabilitation with little supervision has been shown to be as equally effective as rehabilitation with much more supervision following THA and TKA in terms of self-reported function, physical performance and pain ^5, 7^, as reviewed above. So, a natural next step for us is to challenge our own belief that physical rehabilitation (in itself) is important – and surely better than “no physical rehabilitation” – when it comes to enhancing postoperative recovery of functional performance after these surgeries.

In the DRAW 1 trial, we will investigate three different rehabilitation strategies after THA and TKA. We will use two rehabilitation strategies that are commonly used in the community rehabilitation setting in Denmark; home-based telerehabilitation and home-based rehabilitation after initial instruction and compare them to a strategy of no physical rehabilitation as defined in the methods section below. Our primary outcome is the hip disability and osteoarthritis outcome score (HOOS)/knee injury and osteoarthritis outcome score (KOOS)-subscale: function of daily living at the end of the 6-weeks' intervention. This is chosen to be the primary outcome as it is usually physical function that prescribed rehabilitation is targeted to improve ^5, 7^. The primary endpoint is at the 6-week follow up, as it represents current clinical practice in terms of rehabilitation duration for the municipality of Bornholm, Denmark. When we collected stakeholder input for the trial design, the municipality’s main interest in the trial was related to this time point firstly and the other time points secondarily.

### Objective

The objective of the trial is to compare the effectiveness of home-based telerehabilitation, home-based rehabilitation and no physical rehabilitation following THA and TKA.

## Method

### Trial design

This trial is named the “Does rehabilitation after total hip and knee arthroplasty “work”? (DRAW1 trial)”. It uses a superiority, three-arm, parallel group, RCT design with blinded outcome assessments at baseline (before intervention), at 6 weeks (end of intervention) and follow-ups at 3 and 12 months. The trial protocol is based on the PREPARE trial guide ^21^ and the SPIRIT checklist ^22, 23^. The trial report will adhere to the Consolidated Standards of Reporting Trials (CONSORT) using the extension for non-pharmacological treatments ^24^. The interventions will be described in details using the recommended generic template for intervention description and replication (TIDieR) ^25^. For a detailed exercise-specific description of the intervention, the consensus on exercise reporting template (CERT) ^26^ will be used. The trial is pre-registered at ClinicalTrials.gov ( NCT03750448 on 23 ^rd^ November 2018) ^27^ and ethical approvals was obtained from the ethics committee of the Capital Region Denmark ^28^ and the Danish Data Protection Agency ^29^ before the first participant was recruited. The first participant was enrolment in January 2019. The research question, objective, and trial design were developed using stakeholder input from patients, orthopaedic surgeons, physiotherapists and policymakers.

### Pragmatism

The municipality of Bornholm (Bornholms Regional Municipality), where the trial takes place, has been involved in the formulation of the research question and study design. They are considering full-scale implementation of the home-based telerehabilitation-solution based on cost-benefit considerations when compared to their current practice, which is home-based rehabilitation (no tele-setup). The effectiveness of this type of technology-assisted exercise intervention has already been compared to usual care in four municipalities in Copenhagen, Denmark, where comparable effects regarding physical performance and self-reported physical function were seen in both the usual care and telerehabilitation group after a 6-week intervention ^30, 31^. While usual care consisted of supervised exercises twice weekly during 6 weeks (12 supervised exercise sessions in total), the telerehabilitation intervention received 6 supervised exercise classes during the 6-week intervention, limiting what conclusions can be drawn regarding the true efficacy of telerehabilitation. Before investing in the telerehabilitation technology, the municipality of Bornholm wants an estimate of the effectiveness of telerehabilitation during a 6-week intervention *without* supervised exercise sessions to explore the full potential of telerehabilitation (please see the description of the different interventions below).

### Study setting and recruitment

All patients will be included by consecutive sampling from three outpatient rehabilitation trial sites on the isle of Bornholm, Denmark. This consecutive sampling, along with the use of few eligibility criteria, will ensure generalizability of the results. All patients, who receive a THA or TKA and reside on the island, are referred to postoperative, free-of-charge rehabilitation at our institution, reflecting the current clinical practice in Eastern Denmark for patients undergoing THA or TKA. Patients referred to our institution will receive a letter of invitation to initiate outpatient rehabilitation following their surgery ^23^. Written trial information and the patients’ rights as a potential trial participant will be included in this letter, including the right to have a bystander present during the first consultation at our rehabilitation centre.

Patients deemed eligible for study inclusion will be introduced to the trial individually by an experienced physiotherapist, serving as the intervention deliverer, during the first consultation. The consultation will take place in a room with no external interruptions, where patients will be given thorough written and oral information about the nature of the trial by the physiotherapist. Eligible patients will be informed that the trial includes random allocation to one of three intervention groups (home-based telerehabilitation group, home-based rehabilitation group and no physical rehabilitation group) with the purpose of investigating the effectiveness of the three different rehabilitation strategies following THA and TKA. Before deciding to participate in the trial, the patients will be informed about their rights as a trial participant, including the right to have timely consideration before enrolling in the trial. The patients may consider trial participation in the study up to 10 days after they have received oral and written trial information. Patients interested in participating in the trial will be asked to provide written consent according to the template provided by the Ethics Committee of the Capital Region Denmark ^23^. Patients who are willing to participate will sign an informed consent form before any study related procedures are performed. 

To prevent ascertainment bias ^32^, eligible patients are blinded to the trial hypothesis. The physiotherapist attending the first consultation will conduct baseline assessment immediately after the patient has provided written consent and before randomization. All outcome assessors dedicated to the trial will be trained by the primary investigator to ensure standardized outcome assessment and exercise instruction. Patients will be informed that they can, without the need to provide any reason, withdraw their consent and stop their participation in the trial. If written consent is not provided, the patient will be offered usual care at our rehabilitation facility, which is home-based rehabilitation with regular follow-ups.

### Eligibility criteria

In order to increase the external validity and improve the generalizability of this trial, only few eligibility criteria will be used. This will furthermore reflect current clinical practice and enhance the pragmatic nature of the trial.

Inclusion criteria:

- Patients having had primary, unilateral THA or TKA due to osteoarthritis.
- Patients being referred to receive postoperative rehabilitation at our institution.
- Patients being able to speak, read and understand Danish language.
- Patients aged ≥ 18 years.

Exclusion criteria:

- Patients not able to comply with exercise instructions.
- Patients who are discharged to a nursing-home facility or receiving in-home rehabilitation by home care.

### Group allocation

When baseline assessment is completed, the patients will be randomly allocated to one of the three rehabilitation strategies (see assignment of interventions). The attending physiotherapist will then instruct the patients individually according to their allocated group assignment. Once the initial instruction is completed and the patients are comfortable with the intervention to which they are assigned, the session is completed, and no further direct supervision will be given during the 6-week intervention. All patients - no matter their allocation - will receive pamphlets where postoperative information such as recommendations regarding the return to activities of daily living, possible complications and expected discomforts is outlined.

### Interventions

All patients will be given similar pamphlets as mentioned above. The pamphlets will include information of encouragement to stay active as recommended by the Danish National Health Board ^33^ and encouragements to gradually return to activities of daily living. There will be no specific exercise-encouragement given to the no physical rehabilitation group. The two other intervention groups will be prescribed specific exercises for restoring function following THA or TKA. The detailed intervention description and the exercise description are further outlined in our extended data ^23^.

**Figure 1.**
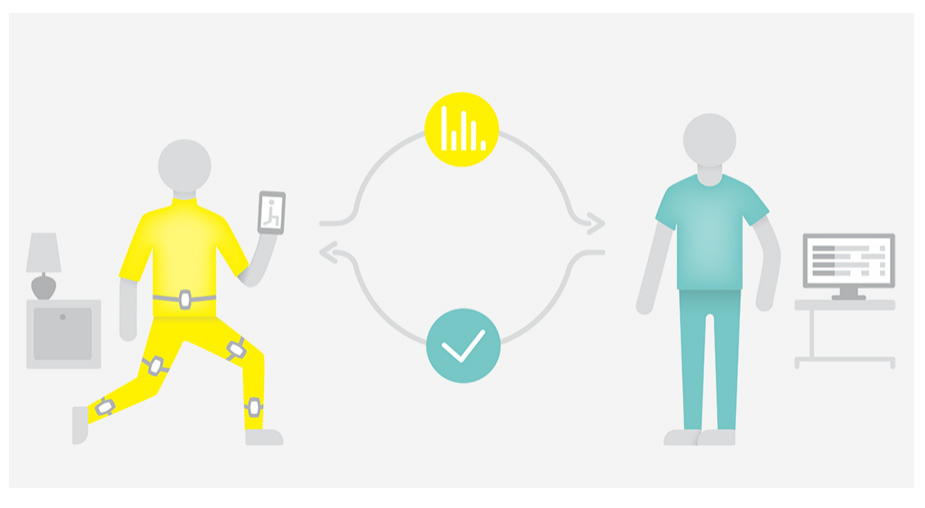
Home-based telerehabilitation using ICURA.

*Home-based telerehabilitation*. Patients randomized to this group will receive interactive virtual rehabilitation using a mobile app. The home-based telerehabilitation is based on sensor technology, developed by ICURA ^34^ ( Figure 1). This technology consists of motion sensors that can measure and analyse the quantity and quality of the exercises, and a mobile application that can guide the patient with visual response. A unique feature of ICURA trainer allows the physiotherapist to remotely supervise the individual patient’s exercise adherence and progress. This technology has already been successfully implemented in several different rehabilitation facilities across Denmark, and, hence, reflects current clinical practice in these places ^34^.

**Figure 2.**
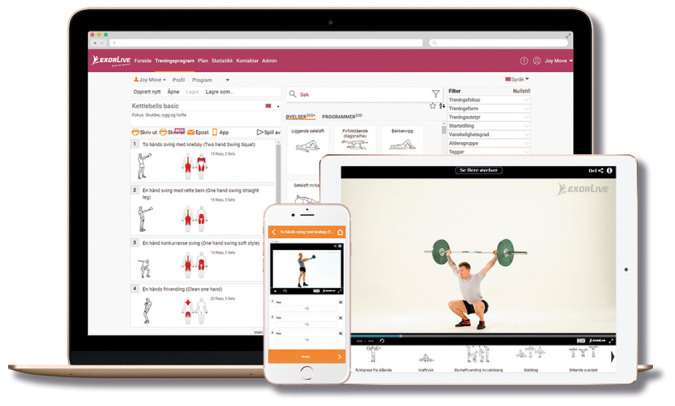
Home-based rehabilitation using Exorlive.

*Home-based rehabilitation.* Patients randomized to this group will be instructed in identical exercises as patients allocated to home-based telerehabilitation. However, this group will receive a written exercise program with instructions on how to perform the exercises at home. The home-based exercise program will be created using exercise templates from Exorlive ( Figure 2). Using a link provided in the exercise program, the patients will be able to see short instruction-videos of the individual exercises.

*No physical rehabilitation.* Patients randomized to this group will not be prescribed any therapeutic rehabilitation exercise. This means that they will not receive any physical activity or exercise designed and prescribed for restoring normal function or reducing pain cause by disease, injury or surgery. This is in contrast to usual care, where exercise instructions are given. Participants in the no physical rehabilitation group will however be encouraged to stay active and continue life as usual, gradually returning to their activities of daily living when they feel ready for it.

### Criteria for discontinuing the allocated intervention

If any patients in any of the allocated groups develop postoperative complications (e.g. postoperative infections, deep venous thrombosis), their participation in the study will be discontinued. The patients will then receive appropriate care and offered outpatient rehabilitation as needed. Study participants will be retained in the trial (unless they withdraw their consent) to enable follow-up data collection and to prevent missing data.

### Criteria for modifying the allocated intervention

Only patients who receive home-based telerehabilitation and home-based rehabilitation are given instructions to modify their allocated intervention. Should any of these patients experience severe hip- or knee symptoms, they will be instructed to lessen the intensity of the prescribed exercise intervention, until the pain has subsided. If this is not sufficient in relieving pain symptoms, the patients are instructed to reduce the volume of the exercise, until the pain has subsided. If the pain persists, the patients are instructed to stop performing the exercises, until the pain has reduced. The patients will then be instructed to gradually progress to the physical intervention to which they are assigned. Detailed, written information will be given to all patients during the first consultation where expected discomfort following THA and TKA will be explained thoroughly. 

### Adherence and concomitant care

During the first consultation, the importance of following the study guidelines and adhering to the allocated group will be highlighted. Exercise adherence will be recorded as a percentage of completed exercises (based on adherence to the prescribed exercise frequency and repetitions) during the first follow up (after the 6-week intervention) for both the home-based telerehabilitation and home-based rehabilitation groups. Exercise adherence will be taken into consideration when performing the per-protocol analysis (please see the statistical methods section).

All enrolled patients will be encouraged to contact the principal investigator directly, if experiencing problems related to their recent surgery or to their allocated group intervention. The principal investigator will then fill out a standardized form at these calls. Patients can use pain-relief products as needed, prescribed by their physician after their surgery. To avoid study-contamination, patients will be asked to adhere to the allocated rehabilitation intervention and not to seek alternative health-care services specific to their hip- or knee arthroplasty during the course of the study. Patients will be advised to contact their general practitioner or the principal investigator as needed.

### Outcomes

This trial is designed with four outcome assessments:

- At baseline (t _0_), during the first consultation following the patient’s THA or TKA (usually between 5 and 7 days postoperatively).
- After the 6-week intervention (t _1_), at our outpatient rehabilitation facility.
- 3 months postoperatively (t _2_), follow-up assessment at our outpatient rehabilitation facility.
- 12 months postoperatively (t _3_), final follow-up assessment at our outpatient rehabilitation facility.

***Primary outcome.*** The primary outcome is the difference between groups in mean score of HOOS/KOOS function in the daily living-subscale (ADL-subscale) at the end of the 6-week intervention. This is chosen as the primary outcome as it is usually physical function that prescribed rehabilitation is targeted to improve ^5, 7^ and therefore the best suited outcome to measure rehabilitation effectiveness. Secondly, restoration of function is often a crucial element of treatment success, which makes this subscale a suitable outcome measure to indicate the restoration of function. The ADL-subscale was identified through semi-structured patient-interviews; patients found the HOOS/KOOS subscale most relevant in order to measure postoperative progress. The outcome will not be calculated as a change score using the baseline value. We do not consider it possible to obtain a valid HOOS/KOOS ADL score before 6 weeks after surgery, because some of the items in the HOOS/KOOS ADL subscale have most often not been sufficiently addressed or experienced by the participants within the first 6 weeks after surgery.

***Secondary outcomes.*** Secondary outcome measurements include the difference between intervention groups during all follow-ups of three subscales from HOOS/KOOS (i.e. pain, symptoms, and quality of life) ^35, 36^. Patient global assessment will be evaluated using a standard question: “How would you rate your current level of function during your usual activities of daily living?” quantified on a 0–100 visual rating scale ^37^. Performance-based assessment will be measured by the 30-s chair stand test, and 40 meters walking test as recommended by the Osteoarthritis Research Society International (OARSI) ^38^. Furthermore, patient satisfaction ^39^ and exercise adherence will be assessed at the end of the 6-week intervention. Current use of analgesics, current use of walking assistant devices and adverse events will be registered within each group (e.g. postoperative infections, deep venous thrombosis or arthrofibrosis) at each follow-up assessment. Outcome assessors will document the time of each individual consultation to investigate the total time of each patient’s rehabilitation and within each of the three rehabilitation strategies. The additional time spent during monitoring of the homebased telerehabilitation intervention will also be documented. 

***Data from medical records.*** All the above-mentioned outcome measurements will be passed on from medical record files in collaboration with the responsible physiotherapist. All data will first be recorded and passed on *after* the patient has provided oral and written consent to participate in the trial.

### Elaborated description of outcome measures

Outcome measurement will adhere to OARSI clinical trials recommendations ^37^.

The patients will complete all questionnaires at each follow up on paper and in individual consultation rooms with no external interruptions. The score of each questionnaire will be calculated by the consulting physiotherapist. The 30s chair-stand-test will be conducted in the consultation room using a test-chair, while the 40 metres walking test will be performed on a test-track near the consultation rooms. All outcome data will finally be reported in the patient’s electronically stored medical record.

***Primary outcome.*** *Hip disability and osteoarthritis outcome score (HOOS) / knee injury and osteoarthritis outcome score (KOOS) subscale: function in daily living (ADL)*. This patient-reported outcome measurement (PROM) has proven to be a valid, reliable and responsive outcome measurement following THA and TKA ^35, 40– 42^. This subscale consists of 17 questions related to the patient’s function in activities of daily living such as “descending stairs”, “standing” and “getting in/out of car”. The subscale is scored by the degree of difficulty that the patient experienced in the last week on a 5-point Likert scale (none, mild, moderate, severe, and extreme) and calculated to a score ranging from 0 (worst/extreme difficulties) to 100 (best/ no problems). The total questionnaire takes about 10 minutes to complete. At least 50% of the questionnaire items are required to be answered to permit calculation of a mean score.

***Secondary outcomes.*** *Remaining three sub-scales of HOOS/KOOS: symptoms, pain, and hip- or knee related quality of life.* A fourth subscale, sport and recreation, was excluded as an outcome measurement at 6 weeks, as most of the items in the HOOS/KOOS sport and recreation subscale often will not have been sufficiently addressed or experienced by the participants within the first 6 weeks after surgery.

Like the above-mentioned subscale, these subscales are scored by the degree of difficulty the patient has experienced in the last week on a 5-point Likert scale. Each subscale provides a score, which can be calculated to a total score ranging from 0 (worst/extreme difficulties) to 100 (best/ no problems). At least 50% of the questionnaire items in each subscale are required to be answered to permit calculation of a mean score. A total score of the questionnaires has not been validated and is therefore not recommended ^36^.

*Patient global assessment.* At baseline the patient’s perception of his or her overall functional ability will be assessed using a single numeric rating scale, as recommended by OARSI ^37^. The patient will be asked a single question: “How would you rate your current level of function during your usual activities of daily living?” During follow-up assessment patients will be asked the same question as at baseline. Their answer will be rated on a 0–100 visual rating scale with end-points anchored as “Inability to perform any daily activities” (0) and “No problem with any daily activity” (100) ^37^.

*30-s chair stand test.* Performance-based assessment will be performed using the 30-s chair-stand test, which represents the *sit-to-stand activity*. The test counts the number of sit-to-stand repetitions the patient can perform in 30 seconds. The straight-back chairs used for testing during all outcome assessments will be of the same model, same height (approximately 43 cm) and will be placed against a wall. Any other adaptations (i.e. the use of armrests or assistive devices) will be reported. To assure understanding, two slow-paced repetitions will be practiced before the test initiates. The 30-s chair stand test has proven good reliability to measure functional performance following THA and TKA ^43, 44^.

*40 meters walking test.* This performance-based assessment measures short distance walking activity. The patient will be instructed to walk as quickly as safely possible to a mark 10 meters away, return and repeat for a total distance of 40 meters. Each walk of 10 meters (excluding the turn time) is recorded and expressed as speed m/s by dividing distance by time in seconds. A practice walk up and back will be performed to assure understanding. Any assistive devices used will be recorded.

*Analgesics.* The use of analgesics will be recorded at baseline and at all follow-up assessments, using a nominal scale (yes/no) regarding the daily consumption of opioids, non-steroid-anti inflammatorily drug (NSAID) and paracetamol. If the patient is not aware of his or her use of analgesics, a medical record will be obtained to assess the use of analgesics. 

*Walking assistant devices.* The type and number (e.g. one or two elbow crutches) will be recorded at baseline and at all follow-up assessments. The need for walking assistant devices will be assessed by the number of walking assistant devices the patient uses during normal activities of daily living.

*Patient satisfaction.* Patient satisfaction will be assessed at the end of the 6-week intervention, where patients will be asked to answer four questions to indicate their level of satisfaction. The answers will be based on a 4-Likert ordinal scale with the categories: very satisfied (100 points), somewhat satisfied (75 points), somewhat dissatisfied (50 points), and very dissatisfied (25 points). The score is the unweighted mean of the combined scores. The four questions are: “How satisfied are you with the results of your surgery and rehabilitation?”, “How satisfied are you with your surgery and rehabilitation for improving your pain?”, “How satisfied are you with the results of your surgery and rehabilitation for improving your ability to do home or yard work?” and “How satisfied are you with the results of surgery and rehabilitation for improving your ability to do recreational activities?”. The questions are modified from the self-administered patient satisfaction scale for primary hip and knee arthroplasty ^39^ and have in its original form proven to be a valid and reliable outcome measurement for patient satisfaction following THA and TKA ^39^.

*Exercise adherence.* Exercise adherence will be measured as a percentage (0–100), where 0 % indicates total non-adherence and 100 % indicates total adherence to the exercise prescription. Exercise adherence in the home-based telerehabilitation and home-based rehabilitation group will be measured as a self-reported outcome, where patients will be asked: “What percentage of the prescribed 42 exercise sessions (one exercise session per day for 6 weeks; corresponding to 100 %) have you completed the past 6 weeks?”

*Adverse events.* Adverse event during enrolment of the trial will be recorded, regardless of the relation to the trial.

*Time usage.* We wish to explore the economic resources required for each of the three rehabilitation strategies. We intend to use this data in our cost-benefit considerations compared to the current practise (home-based rehabilitation).

### Assignment of interventions

Patients deemed eligible for study participation and provided written consent will be randomly assigned to one of the three interventions, after the completion of baseline assessment. A physiotherapist, not otherwise involved in the study, will perform a computer-generated random allocation sequence (1:1:1 allocation rate) concealed in 168 consecutively numbered, opaque, sealed envelopes determining allocation to home-based telerehabilitation, home-based rehabilitation or no physical rehabilitation. Blocked randomisation will be used to achieve balanced group allocations and to ensure concealment; the block sizes will not be disclosed.

### Blinding

Before intervention allocation, baseline measures will be performed and recorded. Experienced physiotherapists, who are blinded to group allocation, will record the follow-up outcome measurements (t _1_ + t _2_ + t _3_) at our outpatient facilities. The patients will be asked not to disclose any information that could give away the group allocation, and the physiotherapist will be instructed not to ask questions that could be used to identify the allocated group of the patient, before follow-up assessment has taken place.

During the following outcome assessment, a different physiotherapist will record the outcome assessment to keep the outcome assessor blinded to group allocation at all times. The patients are only blinded to the trial hypothesis in order to prevent ascertainment bias. The principal investigator will not be blinded, as he does not conduct any outcome assessments, and therefore no emergency unblinding procedure will be necessary.

### Data collection and management

The outcome assessor will record data (patient demographics, use of analgesics, walking assistant devices and possible adverse events) and obtain the HOOS or KOOS questionnaire from the patient on a standardized form during the first consultation, which will take place approximately 3–7 days after discharge. The performance-based outcome assessments will be performed thereafter and also recorded on the standardized form. This procedure will be repeated at the following follow-ups by different physiotherapists to keep outcome assessors blinded for group allocation. Four questions regarding patient satisfaction will be included in the online questionnaire before the 6-week follow-up.

All original written information and case report forms will be stored in a secured room and kept for three years after the trial completion. Electronic data will be anonymized, coded and saved on a secured server in the Capital Region of Denmark, Bornholm. A complete backup of the data entries will be performed every month until trial completion.

Exercise data from the home-based telerehabilitation will be coded and saved on a secured server in the Capital Region of Denmark. The stored data set will be coded in a way so that group allocation is concealed, allowing for blinding of the data analyst.

Participant retention and completion of follow-up assessments are expected to be relatively high due to the comprehensive follow-up assessment of this trial. While standard of care usually includes a 3 months’ follow up, patients included in this trial are invited to follow-up assessments at 6 weeks, 3 and 12 months postoperatively, which includes a free-of-charge consultation with a physiotherapist.

## Statistical methods

The main analysis plan is outlined below, while a detailed analysis can be found in our extended data ^23^.

The trial is designed to test the hypothesis that physical rehabilitation (home-based telerehabilitation and home-based rehabilitation combined) is superior to no physical rehabilitation following THA and TKA or if one of the physical rehabilitation strategies is superior to no physical rehabilitation. Superiority of physical rehabilitation over no physical rehabilitation will be claimed if the between-group contrast at the 6 weeks follow-up is 10 HOOS/KOOS ADL-subscale points or greater in favour of the physical rehabilitation. Superiority of an individual physical rehabilitation strategy over no physical rehabilitation will be claimed if the same criteria are met and in favour of that physical rehabilitation. 

### Analysis outline

The primary outcome is the mean difference in the HOOS/KOOS subscale: function in daily living (ADL). Primary endpoint will be differences between groups at first follow up (t _1_), secondary endpoints being differences at second follow-up (t _2_) and final follow up (t _3_) (see extended data item 3: schedule for enrolment, intervention, and outcome assessments ^23^).

Data analysis will follow the intention-to-treat principle. To allow for full data analysis, missing data will be imputed using multiple imputations.

### Primary analysis

The primary analysis will evaluate the mean difference at 6 weeks follow-up between physical rehabilitation (home-based telerehabilitation and home-based rehabilitation) and no physical rehabilitation. This will be assessed by independent two-sample t-tests if normality assumptions are acceptable, otherwise the Wilcoxon sum rank-test will be used. The same tests will also be used to compare the individual physical rehabilitations to no physical rehabilitation.

### Secondary analysis

The secondary analysis will test differences in the mean score of all continuous secondary outcomes at the primary, secondary and final endpoints and will be performed in the same way as the primary analysis. Nominal outcomes will be analysed by the chi-squared test or in cases with less than five expected counts for any observation, Fishers exact test will be used.

At baseline descriptive statistics will be presented. At each endpoint, mean scores, standard deviations, between-groups mean (with corresponding 95% confidence intervals) and p-values will be reported (see extended data item 4: analysis outline for primary and secondary analysis ^23^).

### Additional analyses

We will undertake exploratory subgroup analyses with stratification on THA or TKA similar to that outlined for the non-stratified analysis.

### Definition of analysis population relating to protocol non-adherence

All analyses will follow the intention-to-treat (ITT) principle (analysed as randomized). Additionally, per protocol (PP) analysis will be done for the primary outcome. All patients with an exercise adherence of at least 80 % will be considered adherent to the rehabilitation strategy and included in the PP analysis. All patients in the no rehabilitation group will be included in the PP analysis. Missing values in both ITT and PP analysis will be imputed by multiple imputation. Imputation models will be based on all other available variables in the data.

### Data monitoring and auditing

No data monitoring committee will be composed, as the interventions pose little or no known risk for the participating patients. Likewise, no auditing procedures are expected to take place.

### Sample size

A clinically important difference of 10 points on the HOOS/KOOS subscale function in daily living (ADL) ^36, 45^ is used as our superiority margin, with an expected standard deviation of 20 points, a power of 0.80 and a significance level of 0.05 resulting in a sample size of 50 patients in each group. With 10% loss to follow up ^46^ this results in 56 patients in each group. This calculation will be used for each comparison of individual physical rehabilitations to no physical rehabilitation; therefore, 56 patients are needed for each of the three groups resulting in 168 patients included in total (3 groups of 56 patients). Comparison of any physical rehabilitation to no physical rehabilitation is done under the same clinically important difference and expected standard deviation as the individual comparisons. With a sample size of 100 (patients receiving one of the two physical rehabilitation strategies) and 50 (patients receiving no physical rehabilitation) the expected power is 0.89.

We expect a relatively low inclusion rate of 50%, since one of the interventions includes “no physical rehabilitation”, compared to standard rehabilitation. Furthermore, based on the expected patient flow at our trial sites and the need to conduct the trial within a realistic timeframe, we do not initially intend to stratify THA and TKA for subgroup analysis. There will not be any specific strategies for increasing participant enrolment related to this trial.

## Ethics and dissemination

### Ethical approval and consent

This trial obtained approvals from the Ethics Committee of the Capital Region Denmark ^28^ and the Danish Data Protection Agency ^29^ and was registered in a public trial registry before the first participant was enrolled. Written consent from each trial participant will be obtained during the first consultation at our outpatient facility by an experienced physiotherapist.

### Confidentiality

All study-related information about the participants will be stored securely on the study site. Paper forms will in stored in locked cabinets, while electronic data will be stored on a password protected drive at the study site. Only persons involved in the trial will have access to the study-related information. Individual study information will not be released outside of the study without permission of the individual participant. This study will be handled in accordance with the Data Protection Act from the Danish Protection Agency.

### Ancillary and post-trial care

It is not expected that the allocated interventions will cause any harm to the participants. If any harm is caused, at the end of the 6-week intervention, the patients will be offered free-of-charge in- or outpatient rehabilitation as deemed clinically needed.

### Access to the trial set

The principal investigator (TMC) and the principal supervisor (TB) will have full access to the data set. The full-anonymized data set will be made available for the journal reviewing the manuscript.

### Dissemination policy

The trial is planned to be reported in four manuscripts and expected to be published in international, peer-reviewed journals. The first manuscript will be the trial protocol. The second manuscript will be the primary report on the investigated effectiveness of the three rehabilitation strategies under investigation. The third manuscript will report the result of the trial from an economic perspective, while the fourth manuscript will report exploratory analyses depending on the primary trial findings. All results, including positive, negative or inconclusive results, will be presented at relevant scientific conferences and symposiums.

All patients will be offered to receive an information letter with the results of this trial when the trial is completed and published. Trial data can be requested by contacting the principal investigator.

**Study status:** Active, not recruiting. Participant no. 168 was recruited on the 27 ^th^ of January 2021. The final follow-up data are expected to be obtained by January 2022.

## Discussion

In 2018, over 10,000 THAs ^47^ and over 9,000 TKAs ^48^ were performed in Denmark. The number of these procedures is expected to increase ^49, 50^, which puts further emphasis on the importance of finding the optimal rehabilitation strategy for this group of patients. As there are significant variations in the rehabilitation practices following THA and TKA both nationally and internationally, investigations of different rehabilitation strategies and their effectiveness are warranted.

Using the FINER-criteria ^51^ we consider the research question feasible, interesting, novel, ethical and relevant. We believe this clinical trial to be highly useful, as we have conducted a thorough literature review of the problem base, included context placement and information gain in the background section, according to the essay published by Ioannidis in 2016 ^52^. Additionally, with careful methodological considerations such as few exclusion criteria and PROM’s we have also ensured a pragmatic and patient-centred trial ^52^. As we will pre-register the trial; use the TIDieR-framework for intervention description ^25^ and publish the trial protocol, we consider the trial design and intent to be as transparent as possible.

In summary, the objective of this trial is to investigate the effectiveness of three different rehabilitation strategies following THA and TKA in terms of function in daily living. Knowledge about the effectiveness of the three investigated rehabilitation strategies will help guide the future organization of post-discharge rehabilitation after THA and TKA.

### Strengths

This trial has several strengths. Firstly, this trial will be the first trial to compare the effectiveness of two commonly used postoperative rehabilitation strategies to a “no physical rehabilitation”-intervention following THA and TKA. Secondly, the trial will be conducted at a geographically ideal setting. The Isle of Bornholm, Denmark, offers a unique opportunity to control study contamination, as our institution is the only one of this kind on the isle. Thirdly, home-based telerehabilitation and home-based rehabilitation-interventions will be given the exact same rehabilitation exercise instructions in terms of exercise type, dosage and intensity, which will make the two physical rehabilitation interventions comparable. Fourthly, randomization and the blinding of outcome assessors and data analysts will greatly decrease the risk of bias of this trial. The consecutive sampling and the relatively unrestricted inclusion criteria increase the external validity.

Lastly, this protocol follows the clinical recommendations from SPIRIT ^22, 23^, CERT ^26^, and TIDieR ^25^, allowing replication and direct clinical use of the described exercise interventions. Furthermore, the adherence to the clinical recommendations from OARSI ^37^ will permit comparison to other trials, eventually allowing meta-analysis.

### Limitations

A noteworthy limitation to this study is the lack of a preoperative outcome assessment. Many patients choose to have their surgery performed at a hospital not located on the island of Bornholm. This means it is not practically possible to obtain preoperative data of these patients before their surgery.

Due to the nature of this trial, there is a risk of selection bias, where active patients will be more likely to participate, compared to inactive patients who are expected to more likely feel the need for supervised rehabilitation. Likewise, the use of a single centre study design represents a common limitation for the external validity of rehabilitation intervention trials. We also anticipate that patient-perceived lack of technical understanding will present some selection bias. However, the current study will nevertheless provide level-one evidence of the effectiveness of the three investigated rehabilitation strategies.

## Data availability

The data referenced by this article are under copyright with the following copyright statement: Copyright: ï¿½ 2021 Mark-Christensen T et al.

Data associated with the article are available under the terms of the Creative Commons Zero "No rights reserved" data waiver (CC0 1.0 Public domain dedication). 

### Underlying data

No data are associated with this article.

### Extended data

Harvard Dataverse: DRAW1. https://doi.org/10.7910/DVN/BUNJQV ^23^

This project contains the following extended data:

- Flowchart of enrolment, randomization, treatment and follow-up
- Schedule for enrolment, intervention and outcome assessments
- Analysis outline for primary and secondary analysis
- Template for intervention description and replication (TIDier) checklist for trial interventions
- Exercise description
- Participant information sheet
- Participant letter of invitation
- Participant consent form

### Reporting guidelines

Harvard Dataverse: DRAW1. https://doi.org/10.7910/DVN/BUNJQV ^23^

This project contains the following reporting guidelines:

- SPIRIT checklist

Data are available under the terms of the Creative Commons Zero "No rights reserved" data waiver (CC0 1.0 Public domain dedication).

### Trial status

**Trial registration**: Clinicaltrials.gov: NCT03750448 (23/11/2018)

**Trial protocol draft version**: 5 (05.01.2021)

**Protocol amendments**: None
