# Supplementary material for: Physical rehabilitation versus no physical rehabilitation after total hip and knee arthroplasties: Protocol for a pragmatic, randomized, controlled, superiority trial (The DRAW1 trial)
Source: F1000Res. 2021 Sep 22;10:146. Originally published 2021 Feb 25. [Version 2] doi: 10.12688/f1000research.50814.2 (PMC8276181; doi:10.12688/f1000research.50814.2)
Supplement: Peer review history

# Peer review: Reviewer response for version 2

**DOI:** 10.5256/f1000research.77649.r95094

**Published:** 2021-09-22

**Authors:** Michael Masaracchio

**Affiliations:** 1 Department of Physical Therapy, Long Island University, Brooklyn, NY, USA

The authors have addressed my comments to my satisfaction. This manuscript is well structured. The authors additionally provided details as to why they chose not to add additional information or content based on my comments. These explanations have satisfied me and I have no other comments to make other than great job.

Is the study design appropriate for the research question?

Yes

Is the rationale for, and objectives of, the study clearly described?

Yes

Are sufficient details of the methods provided to allow replication by others?

Yes

Are the datasets clearly presented in a useable and accessible format?

Not applicable

Reviewer Expertise:

Orthopedic and sports rehabilitation, spinal manipulation for cervical and thoracic spine pathologies and health services research

I confirm that I have read this submission and believe that I have an appropriate level of expertise to confirm that it is of an acceptable scientific standard.

# Peer review: Reviewer response for version 1

**DOI:** 10.5256/f1000research.53902.r86733

**Published:** 2021-07-12

**Authors:** Bayram Unver

**Affiliations:** 1 School of Physical Therapy and Rehabilitation, Dokuz Eylül University, İzmir, Turkey

I criticized the article called "Physical rehabilitation versus no physical rehabilitation after total hip and knee arthroplasties: Protocol for a pragmatic, randomized, controlled, superiority trial (The DRAW1 trial)".

 My opinions are as follows:

- Participants aged ≥18 are specified in the inclusion criteria, but this is a very wide age range, and accordingly, the responses of the participants to rehabilitation vary and affect the results of the study. The lack of preoperative evaluations also contributes to this variability. Therefore, I recommend performing age-related subgroup analyzes or specifying the age of the participants in the inclusion criteria.
- Secondly, participants' involvement in other exercise or treatment programs affects the clinical process. Therefore, 'participating in any program outside of study' should be added to the exclusion criteria.
- The other thing not mentioned in the study is the discharge criteria. Whether or not the same discharge criteria are used for each participant and the details of these criteria contribute to the clarification of the clinical process. Therefore, I recommend including this in the method.
- Since THA and TKA are two different joints and surgeries, rehabilitation procedures and interventions are also different. In the study, these two different interventions were not clearly explained. The type, intensity and dosage of planned interventions or exercises should be detailed.

Is the study design appropriate for the research question?

Yes

Is the rationale for, and objectives of, the study clearly described?

Yes

Are sufficient details of the methods provided to allow replication by others?

Partly

Are the datasets clearly presented in a useable and accessible format?

Partly

Reviewer Expertise:

Orthopedic Physiotherapy and Rehabilitation

I confirm that I have read this submission and believe that I have an appropriate level of expertise to confirm that it is of an acceptable scientific standard.

# Response

**Published:** 2021-09-15

**Authors:** Troels Mark-Christensen

**Affiliations:** Centre of Health, Department of Rehabilitation, Bornholms Regionskommune, Denmark

We would like to thank the reviewer for the helpful comments provided. We found the comments to be fair and the provided input to be very valuable. Our response to the comments are as follows:

**Reviewer 3, comment 1:** I criticized the article called "Physical rehabilitation versus no physical rehabilitation after total hip and knee arthroplasties: Protocol for a pragmatic, randomized, controlled, superiority trial (The DRAW1 trial)".

 Participants aged ≥18 are specified in the inclusion criteria, but this is a very wide age range, and accordingly, the responses of the participants to rehabilitation vary and affect the results of the study. The lack of preoperative evaluations also contributes to this variability. Therefore, I recommend performing age-related subgroup analyzes or specifying the age of the participants in the inclusion criteria.

**Author response:** We initially planned to have very wide, pragmatic inclusion criteria, but we do appreciate and acknowledge the reviewers comment on the inclusion criteria. We will therefore, as recommended, perform an age-relate subgroup analysis.

**Action taken:** Sentence added on page 13, line 422-423: “As the age of the participants may vary and affect the results of the study, we will undertake an age-related subgroup analysis.”

**Reviewer 3, comment 2:** Secondly, participants' involvement in other exercise or treatment programs affects the clinical process. Therefore, 'participating in any program outside of study' should be added to the exclusion criteria.

**Author response:** We do very much agree with the reviewers comment on participants involvement in other exercise/treatments, which may affect the clinical process. We do however ask participant to adhere to the allocated rehabilitation intervention and not to seek other health-care services specific to their THA or TKA during the course of the study (p. 8, line 205-209) and to contact their general practitioner or study principle as needed. Furthermore, one of the exclusion criteria of the trial is: “(…) receiving in-home rehabilitation by home care”. We believe this is the most pragmatic way to avoid study contamination and improve adherence to the study protocol. Lastly, as patient recruitment is finished, no alterations can be applied to the experimental procedures of the trial

**Action taken:** No action taken.

**Reviewer 3, comment 3:** The other thing not mentioned in the study is the discharge criteria. Whether or not the same discharge criteria are used for each participant and the details of these criteria contribute to the clarification of the clinical process. Therefore, I recommend including this in the method.

**Author response:** We do agree with the reviewer’s comment. In Denmark the discharge criteria follow a fast-track pathway, which is standardised across the country. Discharge criteria are: obtainment of basic mobility (independent in transfer and walking with appropriate walking assistant) and acceptable pain levels.

**Action taken:** Sentence added on page 5, line 95-97: “Before discharge from the hospital, patients with hip- or knee arthroplasties have obtained independent basic mobility (transfer and walking) with appropriate walking assistant and experiencing acceptable pain levels.”

**Reviewer 3, comment 4:** Since THA and TKA are two different joints and surgeries, rehabilitation procedures and interventions are also different. In the study, these two different interventions were not clearly explained. The type, intensity and dosage of planned interventions or exercises should be detailed.

**Author response:** The different rehabilitation procedures and interventions are described in detail in extended data, including the type, intensity and dosage.

**Action taken:** No action taken.

# Peer review: Reviewer response for version 1

**DOI:** 10.5256/f1000research.53902.r85826

**Published:** 2021-06-15

**Authors:** Thomas W. Wainwright, Louise Burgess

**Affiliations:** 1 Orthopaedic Research Institute, Bournemouth University, Bournemouth, UK

Thank you for the opportunity to review this well-written study protocol. The protocol utilises reporting guidelines appropriately, and as such is thorough and transparent. 

 The research question and aim is valid and needed, and we agree that to our knowledge this will be the first to compare the effectiveness of physical rehabilitation (either home-based tele-rehab or home-based self guided rehab) vs no physical rehabilitation. 

 We have the following general points that we encourage the authors to consider when reporting the outcomes and drawing conclusions from this study (we note that patient recruitment has already finished and so the experimental procedures will not be able to be altered).

 Primary outcome measure - Use of the KOOS/HOOS is valid and justified by the authors, but please can it's use as the primary outcome measure instead of performance based function tests (which are secondary outcome measures) be explained in light of research that shows early functional recovery post TKA/THA does not always correlate with PROMs (e.g. Luna *et al.*, 2017 ^1^).

 Time usage (or health economic) outcome measure - Within the rationale for the study, the authors explain the need for the local health service to understand the cost benefits/comparisons of the interventions. We feel that more explanation of how the economic analysis will be performed is required. Do the authors intend to include a health resource use measure in addition to comparing physio time? Such health resource questionnaires can account for total related costs of interventions, experienced by both the health service and the personal health related costs of the individual patient. 

 The inclusion of both THA and TKA - From a pragmatic and recruitment perspective we understand why they are grouped together. However, these are two different procedures on different joints with different rehab needs. The early functional recovery (at 6 weeks) of THA and TKA patients can differ, therefore we feel there is a strong need to include a sub-group analysis. From our reading of the text, it is not clear to us whether this is planned or not, for example on page 8 in the "additional analysis" section a subgroup analysis with stratification is discussed, but on page 9 in the "sample size" section it says that an initial subgroup analysis is not planned. Please can this be clarified?

 In summary, this is a well-designed study, and I look forward to learning the findings, and implications for future research and clinical practice. We hope our feedback is useful.

Is the study design appropriate for the research question?

Yes

Is the rationale for, and objectives of, the study clearly described?

Yes

Are sufficient details of the methods provided to allow replication by others?

Yes

Are the datasets clearly presented in a useable and accessible format?

Not applicable

Reviewer Expertise:

Physiotherapy, Rehabilitation, Orthopaedics, Exercise Therapy

We confirm that we have read this submission and believe that we have an appropriate level of expertise to confirm that it is of an acceptable scientific standard.

## References

[1] : Early patient-reported outcomes versus objective function after total hip and knee arthroplasty: a prospective cohort study.*Bone Joint J*.2017;99-B(9) : 10.1302/0301-620X.99B9.BJJ-2016-1343.R11167-117510.1302/0301-620X.99B9.BJJ-2016-1343.R128860396

# Response

**Published:** 2021-09-15

**Authors:** Troels Mark-Christensen

**Affiliations:** Centre of Health, Department of Rehabilitation, Bornholms Regionskommune, Denmark

We would like to thank the reviewers for the helpful comments provided. We found the comments to be fair and the provided input to be very valuable. Our response to the comments are as follows:

**Reviewer 2, comment 1:** Thank you for the opportunity to review this well-written study protocol. The protocol utilises reporting guidelines appropriately, and as such is thorough and transparent. 

 The research question and aim is valid and needed, and we agree that to our knowledge this will be the first to compare the effectiveness of physical rehabilitation (either home-based tele-rehab or home-based self guided rehab) vs no physical rehabilitation. 

 We have the following general points that we encourage the authors to consider when reporting the outcomes and drawing conclusions from this study (we note that patient recruitment has already finished and so the experimental procedures will not be able to be altered)Primary outcome measure - Use of the KOOS/HOOS is valid and justified by the authors, but please can it's use as the primary outcome measure instead of performance based function tests (which are secondary outcome measures) be explained in light of research that shows early functional recovery post TKA/THA does not always correlate with PROMs (e.g. Luna *et al.*, 2017 ^1^).

**Author response:** We agree with the reviewer’s comment, that performance-based outcomes does not always correlate with PROMS and that both outcome assessments are relevant, which is why we also use both kind of assessments. We chose to use PROM as the primary outcome measurement, as this outcome measurement was determined as the most appropriate primary outcome by the stakeholders during the planning of the trial, and we have now highlighted this in the protocol. Furthermore, the use of a PRO instrument is advised when measuring a concept best known by the patient or best measured from the patient perspective ( Guidance for Industry (fda.gov).

**Action taken:** No action taken.

**Reviewer 2, comment 2:** Time usage (or health economic) outcome measure - Within the rationale for the study, the authors explain the need for the local health service to understand the cost benefits/comparisons of the interventions. We feel that more explanation of how the economic analysis will be performed is required. Do the authors intend to include a health resource use measure in addition to comparing physio time? Such health resource questionnaires can account for total related costs of interventions, experienced by both the health service and the personal health related costs of the individual patient. 

**Author response:** Thank you very much for bringing up this issue. The municipality intends to investigate health resource use by taking the physiotherapists time into account. No other resource use item is intended to be applied. We do, however, acknowledge the need for a more thorough economic assessment of the interventions applied, if the primary aim of this trial was in a health economic perspective. As patient recruitment is finished, no alterations can be applied to the experimental procedures of the trial. However, in hindsight, additional health resource questionnaires should have been included.    

**Action taken:** No action taken.

**Reviewer 2, comment 3:** The inclusion of both THA and TKA - From a pragmatic and recruitment perspective we understand why they are grouped together. However, these are two different procedures on different joints with different rehab needs. The early functional recovery (at 6 weeks) of THA and TKA patients can differ, therefore we feel there is a strong need to include a sub-group analysis. From our reading of the text, it is not clear to us whether this is planned or not, for example on page 8 in the "additional analysis" section a subgroup analysis with stratification is discussed, but on page 9 in the "sample size" section it says that an initial subgroup analysis is not planned. Please can this be clarified? In summary, this is a well-designed study, and I look forward to learning the findings, and implications for future research and clinical practice. We hope our feedback is useful.

**Author response:** Thank you for bringing this to our attention. We do agree that THA and TKA are different procedures on different joints with different rehab needs, which is why we also intent to include a subgroup analysis between THA and TKA. As the initial sample size does not allow for stratified analysis, it was our intention to double the sample size of the trial by continuing the study setup in a supplementary trial if possible. After the trial inclusion was completed and the target sample size was obtained, an opportunity to continue the trial were presented and at the same time the municipality asked for stratified data to help guide their rehabilitation practice. This first trial (DRAW1) will therefore not include stratified analyses, while a following trial (DRAW2) eventually will allow for stratified analysis. A detailed description of DRAW2 can be found here: https://clinicaltrials.gov/ct2/show/NCT04960241?term=mark-christensen&draw=2&rank=1

**Action taken:** Sentence deleted on page 8 (page 13, line 421-422 in MW-document): “We will undertake exploratory subgroup analyses with stratification on THA or TKA similar to that outlined for the non-stratified analysis.”

# Peer review: Reviewer response for version 1

**DOI:** 10.5256/f1000research.53902.r85823

**Published:** 2021-06-02

**Authors:** Michael Masaracchio

**Affiliations:** 1 Department of Physical Therapy, Long Island University, Brooklyn, NY, USA

Thank you for the opportunity to review this important manuscript. Overall, this is one of the better manuscripts I have reviewed in some time. This is an important topic and the writing is concise, clear, and easy to follow. Of course another read-through is always worth it. 

 As this is a protocol, my comments will be specific to the information in this manuscript, however, other comments I make can be applicable to the pending RCT that can have huge implications for physical therapy.

 First, I think the title of this paper needs to clearly specify that this is tele-rehabilitation or home rehabilitation and not simply say rehabilitation. I strongly suggest this as depending on the results we would want all readers to clearly know the type of rehabilitation provided up front, especially since no group is getting formal outpatient physical therapy.

 Second, it appears that the initial PT making the introduction to the patients about the trial has no other role in the study? If this is the case, I would like to see that made more explicitly clear.

 Third, my biggest concern is two fold. First, why was the word rehabilitation used as opposed to physical therapy? Are there going to be providers who are not physical therapists in this study? If the authors want to use the word rehabilitation, I think it is important to operationally define why this was chosen rather than physical therapy. Perhaps, this can be specific to Denmark, but in the US, these terms are not synonymous and can have implications and be interpreted differently. If is specific to Denmark, and the authors feel there would be no miscommunication or confusion, my comment can be ignored. Secondly and more importantly, my concern is the term rehabilitation and how the interventions are being applied. Physical therapy/rehabilitation is not only about exercise. There are other components, such as manual therapy, and motor control/movement training that can be just as important if not more important following THA or TKA. Depending on the results it may be more appropriate to conclude that exercise and education in isolation are no better/better (yes, this needs to be determined with the RCT) than no physical rehabilitation. It is important to clarify this because in the US physical therapy is not only exercise. It is a combination of manual therapy, exercise, and movement training, along with possible modalities at times. It is the clinical reasoning that is important in how these interventions are put together and in what dosage they are prescribed at. 

 Lastly, I would suggest assessing some form of strength measure for the quadriceps and hip abductors as research continues to discuss the long term strength deficits for the quadriceps after TKA and gluteus medius following THA depending on the approach used.

Is the study design appropriate for the research question?

Yes

Is the rationale for, and objectives of, the study clearly described?

Yes

Are sufficient details of the methods provided to allow replication by others?

Yes

Are the datasets clearly presented in a useable and accessible format?

Not applicable

Reviewer Expertise:

Orthopedic and sports rehabilitation, spinal manipulation for cervical and thoracic spine pathologies and health services research

I confirm that I have read this submission and believe that I have an appropriate level of expertise to confirm that it is of an acceptable scientific standard, however I have significant reservations, as outlined above.

# Response

**Published:** 2021-09-15

**Authors:** Troels Mark-Christensen

**Affiliations:** Centre of Health, Department of Rehabilitation, Bornholms Regionskommune, Denmark

We would like to thank the reviewer for the helpful comments provided. We found the comments to be fair and the provided input to be very valuable. Our response to the comments are as follows:

**Reviewer 1, comment 1:** Thank you for the opportunity to review this important manuscript. Overall, this is one of the better manuscripts I have reviewed in some time. This is an important topic and the writing is concise, clear, and easy to follow. Of course another read-through is always worth it. 

 As this is a protocol, my comments will be specific to the information in this manuscript, however, other comments I make can be applicable to the pending RCT that can have huge implications for physical therapy. I think the title of this paper needs to clearly specify that this is tele-rehabilitation or home rehabilitation and not simply say rehabilitation. I strongly suggest this as depending on the results we would want all readers to clearly know the type of rehabilitation provided up front, especially since no group is getting formal outpatient physical therapy.

**Author response:** We initially want to emphasize that the trial was designed firstly as a superiority trial for the comparison of physical rehabilitation versus no physical rehabilitation, which is also reflected in the pre-defined plan for the analyses. This is the main focus and rationale of trial and, hence, what we want to emphasize transparently in the title. We do, however, clarify the different types of rehabilitation provided in the abstract so that anyone who does a title/abstract screen will see this. We hope the reviewer can appreciate the reasons for this choice.

**Action taken:** None

**Reviewer 1, comment 2:** Second, it appears that the initial PT making the introduction to the patients about the trial has no other role in the study? If this is the case, I would like to see that made more explicitly clear. 

**Author response:** We agree. The introducing PTs role is to conduct baseline assessment first and deliverer of intervention second, and should be explicitly stated.

**Action taken:** Sentence added on page 6, line 119-121: “Baseline assessment is performed before randomization and the allocated intervention is reveal to the patient and the attending physiotherapist”. Sentence added on page 6, line 121-122: “All outcome assessors dedicated to the trial are licensed physiotherapists”. Lastly, sentence added on page 6, line 126-129: “After the baseline assessment has been performed and information about the allocated intervention has been provided, the consultation ends and the attending physiotherapist who conducted the baseline outcome assessment has no further role in the study. 

**Reviewer 1, comment 3:** Third, my biggest concern is two fold. First, why was the word rehabilitation used as opposed to physical therapy? Are there going to be providers who are not physical therapists in this study? If the authors want to use the word rehabilitation, I think it is important to operationally define why this was chosen rather than physical therapy. Perhaps, this can be specific to Denmark, but in the US, these terms are not synonymous and can have implications and be interpreted differently. If is specific to Denmark, and the authors feel there would be no miscommunication or confusion, my comment can be ignored

**Author response: **In Denmark, hospital discharge following THA or TKA is combined with a postoperative “rehabilitation plan” which is essentially a prescription of an outpatient health care service (physical rehabilitation) to be delivered in the patient’s own municipality. In most cases, the service deliverer are physical therapists. There are, however, regional differences in how that municipality rehabilitation is organized and constructed - but it reflects the context-specific interpretation of the available scientific evidence. It typically includes both exercise and non-exercise components. Because the DRAW1 trial is a pragmatic trial, we have made no efforts to modify current clinical practice, as it represents “real (clinical) life” in one municipality in Denmark. We simply ask if the commonly used practice of a post-operative rehabilitation plan and corresponding out-patient physical rehabilitation in the patient’s own municipality is superior to no physical rehabilitation. So, to reflect this organization of health care service and the terms used to describe if, we think “physical rehabilitation” is the best fit.  

**Action taken:** We have added a section on page 4, sentence 47-51 outlining parts of the answer above: “Because the DRAW1 trial is a pragmatic trial, we have made no efforts to modify current clinical practice, as it represents “real (clinical) life” and interpretation of the available scientific evidence in one municipality in Denmark. We ask if the commonly used practice of a post-operative rehabilitation plan and corresponding out-patient physical rehabilitation in the patient’s own municipality is superior to no physical rehabilitation”.

**Reviewer 1, comment 4:** Secondly and more importantly, my concern is the term rehabilitation and how the interventions are being applied. Physical therapy/rehabilitation is not only about exercise. There are other components, such as manual therapy, and motor control/movement training that can be just as important if not more important following THA or TKA. Depending on the results it may be more appropriate to conclude that exercise and education in isolation are no better/better (yes, this needs to be determined with the RCT) than no physical rehabilitation. It is important to clarify this because in the US physical therapy is not only exercise. It is a combination of manual therapy, exercise, and movement training, along with possible modalities at times. It is the clinical reasoning that is important in how these interventions are put together and in what dosage they are prescribed at. 

**Author response: **In our response to comment 3 above, we provide our reasoning for the choice of wording. We do not agree that particular components of physical therapy/rehabilitation or single modalities offer superior effects in the rehabilitation efforts following THA or TKA, neither on short nor long term. The supporting evidence is now quite substantial ( https://pubmed.ncbi.nlm.nih.gov/24287215/, https://pubmed.ncbi.nlm.nih.gov/25886975/, https://pubmed.ncbi.nlm.nih.gov/27584144/, https://pubmed.ncbi.nlm.nih.gov/27401004/, https://pubmed.ncbi.nlm.nih.gov/25724323/).  As physical rehabilitation in the form of physical activity and exercise prescription is currently recommended despite its lack of clear superiority over a range of different comparators, we have decided to investigate if such prescription is superior to no prescription of physical rehabilitation

**Action taken:** No action taken.  

**Reviewer 1, comment 5:** Lastly, I would suggest assessing some form of strength measure for the quadriceps and hip abductors as research continues to discuss the long term strength deficits for the quadriceps after TKA and gluteus medius following THA depending on the approach used.

**Author response:** We did consider to include some form of strength measure, but since we wanted to design a pragmatic trial with a simple outcome assessment (to increase uptake and fidelity). As patients are concerned about their functional performance, and not their strength (even though is can be used as a surrogate outcome measure), we prioritized a functional assessment. And finally, as recruitment has been concluded, inclusion of another outcome measure is not possible. 

**Action taken:** No action taken.
